# Spatiotemporal trends in *P. falciparum* malaria and identification of high-risk villages in Eastern Myanmar: an 8-year observational study

**DOI:** 10.1038/s41598-025-32065-z

**Published:** 2025-12-11

**Authors:** Jade D. Rae, Daniel M. Parker, Richard J. Maude, Aung Myint Thu, Angela Devine, Francois H. Nosten, Julie A. Simpson

**Affiliations:** 1 Shoklo Malaria Research Unit (SMRU), Mahidol-Oxford Tropical Medicine Research Unit (MORU), Faculty of Tropical Medicine, Mahidol University, Mae Ramat, Thailand; 2 Mahidol-Oxford Tropical Medicine Research Unit (MORU), Faculty of Tropical Medicine, Mahidol University, Bangkok, Thailand; 3 Research Group Neglected Diseases and Envenoming, Bernhard Nocht Institute for Tropical Medicine (BNITM), Hamburg, Germany; 4 Population Health and Disease Prevention, University of California-Irvine, Irvine, CA USA; 5 Epidemiology and Biostatistics, University of California-Irvine, Irvine, CA USA; 6 Centre for Tropical Medicine and Global Health, Nuffield Department of Medicine, University of Oxford, Oxford, UK; 7 The Open University, Milton Keynes, UK; 8 Centre for Health Policy, Melbourne School of Population and Global Health, The University of Melbourne, Melbourne, Australia; 9 Global and Tropical Health Division, Menzies School of Health Research, Charles Darwin University, Darwin, Australia; 10 Centre for Epidemiology and Biostatistics, Melbourne School of Population and Global Health, The University of Melbourne, Melbourne, Australia

**Keywords:** Malaria, *P. falciparum*, High-risk, Geostatistics, Diseases, Health care, Medical research

## Abstract

One barrier to achieving *Plasmodium falciparum* elimination is the persistence of villages where transmission remains high. While targeted interventions can effectively reduce transmission in these areas, identifying priority target villages is often resource-intensive. This study investigates the use of a geostatistical model to analyse routinely collected surveillance data and identify high-risk villages in Hpapun Township, Myanmar. A geostatistical model was fitted using routine surveillance data (2014–2021) collected from 507 village-based malaria posts to assess temporal changes in *P. falciparum* incidence and make incidence predictions while accounting for elevation, prior interventions and spatial correlation between villages. Between 2014 and 2019, *P. falciparum* incidence decreased by 93.9%. Villages that received targeted interventions were characterised by higher pre-intervention incidence (incidence rate ratio (IRR) = 4.72, 95% confidence interval (CI) 4.56–4.90) relative to non-intervention villages and were associated with lower incidence post-intervention (IRR = 0.26, 95% CI 0.24–0.27). In 2021, 12 high-risk villages were identified, with a reported incidence exceeding the predicted incidence for at least three months, and eight villages were identified as persistently high-risk (≥ 90th percentile difference in at least six months). Our findings suggest that geostatistical modelling can be utilised to identify persistent high-risk villages, thereby efficiently supporting malaria elimination efforts.

**Supplementary Information:**

The online version contains supplementary material available at 10.1038/s41598-025-32065-z.

## Introduction

Malaria transmission is highly spatially heterogeneous. As incidence declines in a given region, this spatial heterogeneity often becomes more pronounced, revealing villages where case numbers remain disproportionately high despite regional declines^1–4^. These villages tend to cluster together due to similar epidemiology^5^, partly attributed to shared environmental conditions, vector ecology, and human movement between neighbouring villages. These villages represent barriers to achieving local elimination, as persistent transmission not only prevents elimination within the village, but also in its surrounding area^4^.

In addition to maintaining early diagnosis and treatment services, targeted interventions may be needed to reduce ongoing transmission in villages with high case numbers. Targeted interventions include mass drug administration (MDA), mass screening and treatment (MSAT)^4,6,7^, vector control measures^8,9^, and community engagement activities^10^. However, because targeted interventions are typically resource-intensive, their delivery should be prioritised to villages with high case numbers that persist over time and where these interventions are likely to have the greatest impact on malaria transmission^11,12^.

There are several approaches for identifying malaria areas with consistently high case numbers. One option is cross-sectional quantitative polymerase chain reaction (qPCR) surveys, which involve collecting blood samples from the consenting adult population of a village and screening them for patent and sub-patent malaria infections. This method provides a reliable estimate of village-level burden^6^, but is resource-intensive and limited to capturing infection prevalence at the time of the survey. Another approach is to use routine, passive surveillance data. Although this is limited to symptomatic individuals who present for malaria testing, data are collected over time at little cost and can be analysed to identify persistent malaria transmission areas^13–15^. Malaria cases can thus be classified based on the detection method. qPCR surveys identify both asymptomatic infections and clinical cases, while passive surveillance detects only clinical cases, capturing the number of infections that seek care and are diagnosed, rather than the full extent of infection.

Since 2014, the Malaria Elimination Task Force (METF) programme has operated a vast network of village-based malaria posts across Karen State, Myanmar, which provide early access to diagnosis and treatment for malaria cases. Following substantial reductions in *Plasmodium falciparum* incidence between 2014 and 2021 in the METF program area, the distribution of *P. falciparum* infections became increasingly heterogeneous, with the majority of cases concentrated in villages located in Hpapun Township, in the northern part of Karen State^16^.

In this retrospective study, weekly passive surveillance data collected at the METF malaria posts in Hpapun Township between 2014 and 2021 were used to construct a geostatistical model of *P. falciparum* monthly incidence. The aim was to identify “high-risk” villages where the reported incidence was persistently higher than model-based predictions over time. These villages may represent areas where transmission patterns deviate from broader spatial patterns and may warrant targeted investigation or intervention.

## Methods

### Study design and setting

**Fig. 1:**
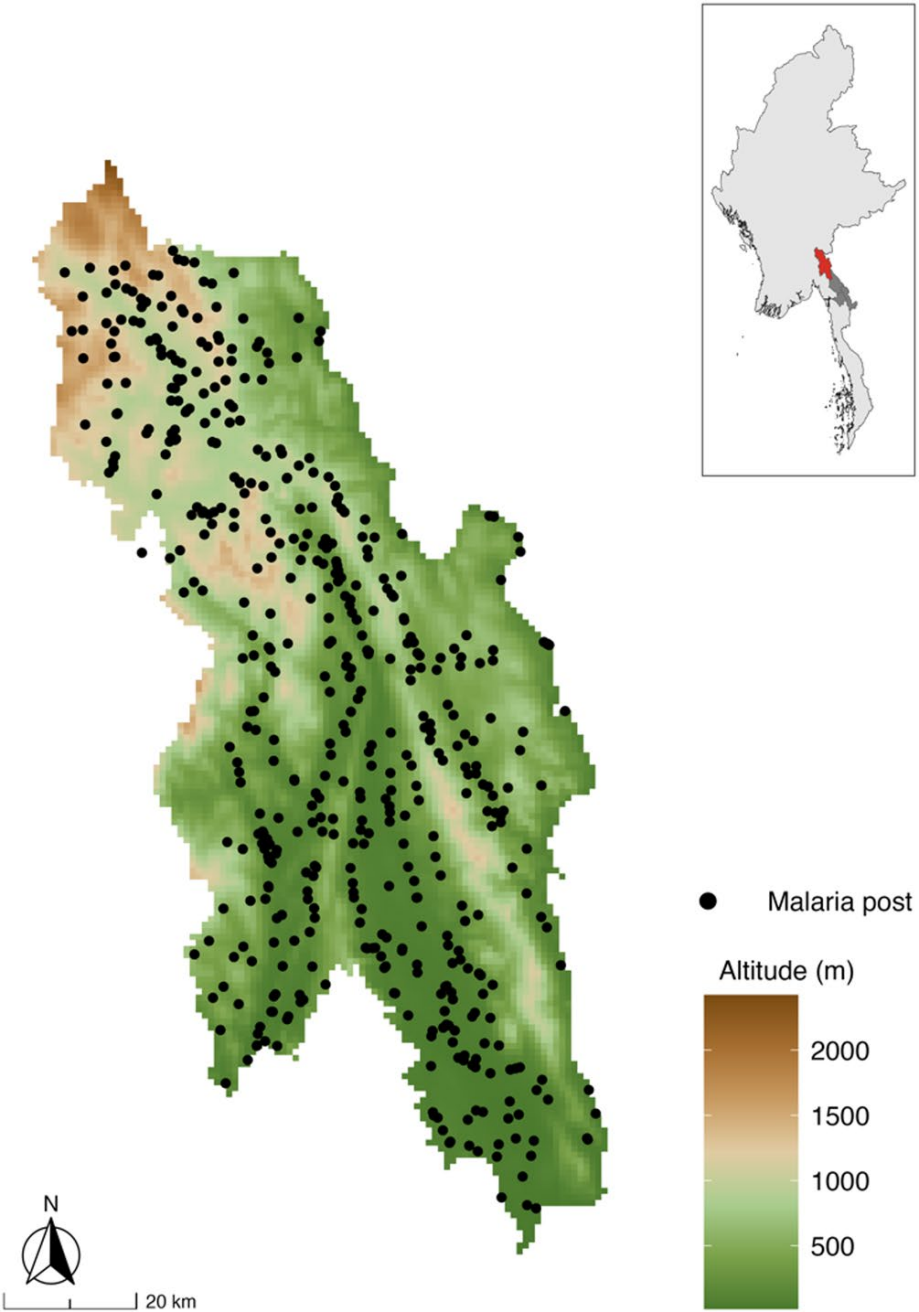
The village-based malaria posts of Hpapun Township and their varying geography. The elevation at the METF malaria posts in Hpapun Township ranges from lowlands (15 m above sea level) to highlands (up to 2,473 m above sea level). The inset map of Myanmar shows the location of Karen State (dark grey) and Hpapun Township (red).

This retrospective, observational study uses weekly surveillance data collected from all 507 village-based malaria posts in Hpapun Township between 2014 and 2021 (Fig. 1). These data were used to identify villages where the monthly *P. falciparum* incidence was persistently higher than expected, based on modelled predictions and the overall declining trend in incidence over time.

Hpapun covers an estimated 6,730 km^2^ of mountainous, heavily forested terrain with limited road networks. There are typically two annual malaria transmission peaks, one at the start of the wet season, from May to July, and another in the cold season, from November to January. During the wet season, travel in Hpapun becomes increasingly difficult due to flooding, landslides, and the faster-moving Salween River, which runs down its eastern boundary.

### Data

#### Weekly surveillance data

Each week, malaria post workers recorded the total number of individuals tested using rapid diagnostic tests (RDTs) and the number of *P. falciparum* or *P. vivax* cases diagnosed, by age and sex. These data were physically transported to the local data entry site, where they were entered into Microsoft Access within 14 days of the end of the reporting week. The electronic records were then sent to the central METF office, where they were merged with data from the previous weeks. Data quality checks were performed routinely to identify inconsistencies and ensure data completeness.

#### Environmental data

The elevation of each village was extracted from elevation raster data for Myanmar (CGIAR-CSI SRTM data obtained from https://diva-gis.org/data.html) and combined with the weekly surveillance data. Other environmental variables, such as vegetation and rainfall, were initially considered in the model. However, their inclusion led to convergence issues due to model complexity. As a result, elevation was used instead, serving as a proxy for associated environmental factors in the area.

#### Intervention data

For the villages where targeted malaria interventions (MDA or MSAT) were delivered, either in response to high prevalence confirmed by qPCR surveys in the case of MDA^6^ or persistently high incidence in the case of MSAT^7^, the date of the intervention was extracted from the METF intervention database. Targeted vector control interventions were sometimes deployed concurrently with MDA or MSAT. This information was merged with the weekly surveillance data to identify villages that had received an intervention and determine which weekly records were before or after the intervention was delivered.

### Statistical analysis

#### Incidence calculation

Monthly *P. falciparum* incidence was calculated from weekly surveillance data as the number of cases each month over person-months exposed for each village. Person-months exposed was calculated at the village level using household numbers collected at the time of malaria post opening^17^ and assuming an estimated population of 5.5 persons per household (based on village census information collected at some malaria posts at the start of the program^18^.

#### Descriptive statistics

Temporal changes in *P. falciparum* incidence were assessed by calculating and comparing the mean monthly incidence between years and within defined periods.

#### Model specification

Poisson mixed-effects modelling was performed to investigate changes in *P. falciparum* incidence over time, with the population in each village per month included as the person-time denominator. To account for the non-linear temporal trend in *P. falciparum* incidence, time in months since May 2014 (when the malaria post network was deployed) was included as a continuous value with a natural cubic spline with two knots at approximately August 2017 and October 2019. Fourier terms were included to account for seasonality in malaria transmission over time. To account for the non-linear association between geography and *P. falciparum* incidence, the elevation of each village was included as a scaled continuous value for meters above sea level, with a natural cubic spline with two knots at approximately 100 and 500 m above sea level. The relationship between *P. falciparum* incidence and elevation is shown in Supplementary File 1, Figure S1. To account for the impact of MDA^6^ and MSAT^7^ on *P. falciparum* incidence, a binary covariate was included for villages that had never or ever received a targeted intervention. Additionally, a binary covariate was included to indicate whether each month occurred before (or, for non-intervention villages, in the absence of) or after the delivery of an intervention. The relationship between *P. falciparum* incidence and intervention delivery is shown in Supplementary File 1, Figure S2. To account for residual variation in *P. falciparum* incidence not captured by the covariates included in the model, a random effect for village was included. Negative binomial regression modelling was also explored, but based on its results, it did not capture the residual spatial correlation in incidence measurements. The Poisson model was fit using the glmer function, and the negative binomial model was fit using the glmer.nb function, from the lme4 package^19^.

Non-spatial models (such as the Poisson mixed-effects model described above) do not account for spatial dependencies between *P. falciparum* incidence measurements over space, i.e., they have a built-in assumption that the incidence at one village does not influence the incidence at a neighbouring village^20^. After fitting the model, an empirical variogram of the residuals from the model was plotted to determine whether this assumption is valid^21^. A description of this validation is provided in Supplementary File 2, and the corresponding results, which indicate the presence of residual spatial correlation, are provided in Supplementary File 2, Figure S3.

To account for the spatial correlation in *P. falciparum* incidence between villages identified by the empirical variogram, the model was extended to include a spatially structured term This spatial component acts as a proxy for the combined effects of the unmeasured explanatory variables that influence *P. falciparum* incidence at a given village, and accommodates additional variation that a standard Poisson model cannot capture, thereby reducing overdispersion attributable to spatially structured heterogeneity. This model is hereafter referred to as the geostatistical model and is described in Eq. 1, Supplementary File 2. The spatially structured residuals were modelled using the exponential correlation function,

$$\rho \left( u \right) = \exp \left( { - u/\phi } \right),$$

where u is the distance between malaria posts and ϕ is the estimated scale parameter. The practical range of spatial correlation is defined as the distance at which correlation falls below 0.05. Solving for ρ(u) = 0.05 gives,

$$u = \phi \ln \left( {20} \right),$$

so the estimated scale parameter ϕ from the geostatistical model was multiplied by ln(20) to obtain the spatial correlation distance^21^. The geostatistical model was fit using all data available from May 2014 to November 2021 using the glgm.LA function in the PrevMap package^22^. A description of the geostatistical model and its validation is provided in Supplementary File 2, and the corresponding results are provided in Supplementary File 2, Figure S4.

#### Spatial prediction

The geostatistical model was used to make out-of-sample predictions of monthly *P. falciparum* incidence at each village between January 2021 and December 2021 using data collected from malaria post opening until the month prior to the prediction month. For example, if a malaria post opened in May 2014, the prediction of *P. falciparum* incidence for December 2021 was made using the geostatistical model fitted to data from May 2014 to November 2021.

The difference between the predicted incidence from the geostatistical model and the reported incidence from the surveillance data was calculated to obtain the fold difference. The fold difference was then mapped to identify villages where (1) the reported incidence exceeded the predicted incidence in a given month, and (2) the reported incidence exceeded the predicted incidence persistently over time. To identify priority villages for targeted intervention, the number of months (between January 2021 and December 2021) in which the fold difference was at or above the 90th percentile (derived from all villages that month) was calculated for each village and referred to as “high-risk” months. A strict 90th percentile cut-off was chosen to identify the most “high-risk” villages in the study period. This cut-off can be adjusted to identify more or fewer villages based on the availability of resources to respond to these locations.

All analyses were performed and all maps were generated using R Statistical Software (version 4.2.1; R Core Team 2022). The boundary shapefile for the Hpapun area used in the maps was obtained from Humanitarian Data Exchange^23^.

### Ethics declarations

The METF programme is approved by the Department of Medical Research (Lower Myanmar) (73/Ethics 2014) and the Tak Community Advisory Board (TCAB-09/REV/2016). All study activities were conducted in accordance with the approved guidelines.

## Results

### Trends in *P. falciparum* incidence

**Fig. 2:**
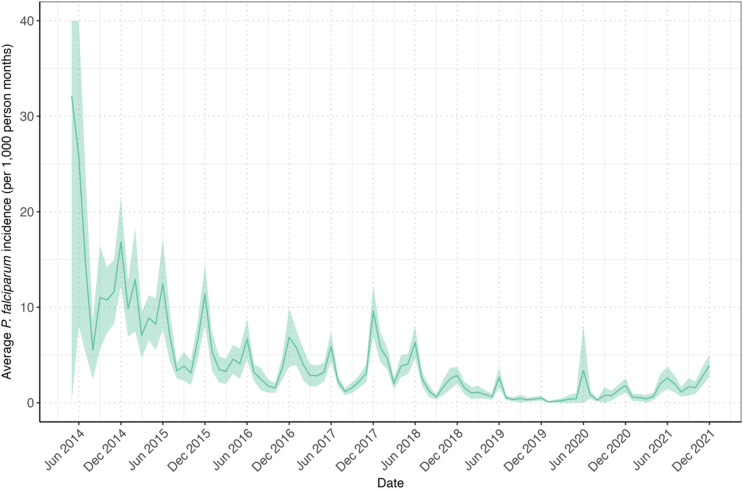
Mean monthly *P. falciparum* incidence in Hpapun Township between May 2014 and December 2021. The mean monthly *P. falciparum* incidence (green line) with 95% confidence intervals (green area) was summarised across the 507 METF malaria posts in Hpapun Township.

Since the beginning of the METF program in 2014, the reported monthly *P. falciparum* incidence has varied across Hpapun Township. From 2018 to 2021, transmission became largely confined to Hpapun Township, where clusters of villages with high incidence emerged^16^. Between 2014 and 2019, the monthly incidence of *P. falciparum* in Hpapun Township declined by 93.9%, from 13.99 cases to 0.86 cases per 1000 person-months (Fig. 2). However, in 2020, the mean monthly incidence rose slightly to 0.88 cases per 1,000 person-months, followed by an increase in 2021 to 1.66 cases per 1,000 person-months.

### Factors influencing *P. falciparum* incidence

In villages where targeted interventions (MDA or MSAT) were deployed, *P. falciparum* incidence was 4.7 times higher (95% confidence interval (CI): 4.56–4.90) compared to villages without such interventions (Supplementary File 2, Table S1). Following a targeted intervention, incidence in these villages decreased considerably, with an incidence rate ratio (IRR) of 0.26 (95% CI: 0.24–0.27) compared to pre-intervention levels or to villages without targeted interventions (Supplementary File 2, Table S1).

The estimated spatial correlation range, derived from the transformed scale parameter (ϕ), was approximately 43 km (95% CI: 37–50 km) and is consistent with previous assessments of spatial correlation in METF villages^6^. Compared with the model without elevation, the scale parameter in the fitted geostatistical model was attenuated by 37% (from 22.79 to 14.33), while the spatially structured variance increased from 0.36 to 0.42, and the nugget variance (τ^2^) increased from 0.12 to 0.17. This suggests that elevation accounts for part of the broad-scale spatial correlation, leaving short-range correlation and localised variation in the residual process. Validation of the geostatistical model indicated good agreement between the fitted model and the data (Supplementary File 2, Figure S4).

### Spatial prediction

**Fig. 3:**
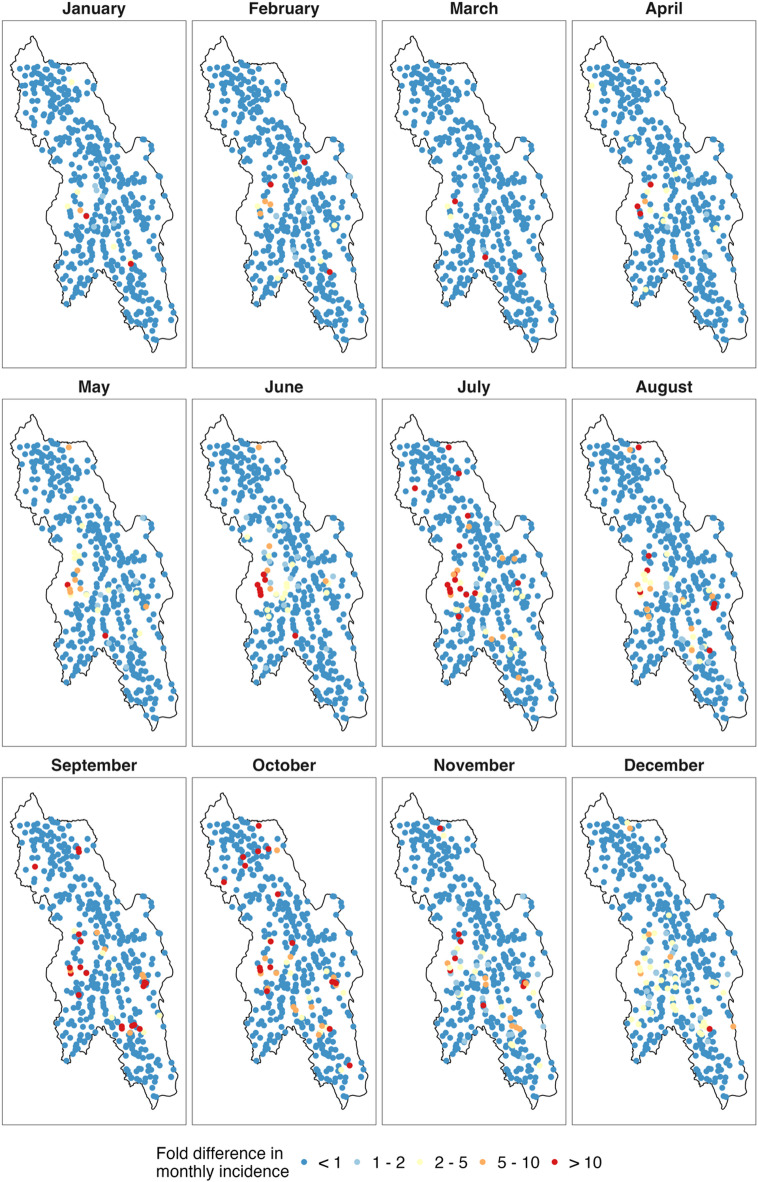
Fold difference between the reported and predicted *P. falciparum* monthly incidence at each village. Fold differences are shown for each village and month in 2021, ranging from dark blue (< 1), indicating a lower reported incidence than predicted, to red (> 10), where the reported incidence greatly exceeded the predicted incidence from the geostatistical model.

Monthly predictions of *P. falciparum* incidence between January and December 2021 from the geostatistical model were compared to the reported incidence for each malaria post. During this period, the majority of reported incidence rates (92.9%, 5168/5562) were lower than the model’s predictions (Fig. 3). In 2021, most malaria post reports (91.7%, 5098/5562) included fewer than one case per 1,000 person-months. By month, this proportion ranged from 82.8% in December to 98.1% in March.

From September to December 2021, there was an increase in the number of malaria posts reporting an incidence two to five times higher than the predicted incidence (yellow malaria posts in Fig. 3). However, these villages were not persistent over 2021 and represent an unexpected increase in *P. falciparum* incidence in December 2021.

On the other hand, 2.8% (158/5,562) of reported monthly incidence rates were at least five times higher than the predicted incidence in 14.4% (71/494) of malaria posts, primarily located in the central west of Hpapun Township (shown as orange and red points in Fig. 3). Additionally, 42 of these malaria posts reported incidences at least 10 times higher than predicted in at least one month. Among these 42 malaria posts, two reported an incidence at least 10 times higher than the predicted incidence in six out of the 12 months, 10 posts in three to five months, and the remaining 30 posts in one to two months (red malaria posts in Fig. 3). An additional table lists the codes of the 12 malaria posts with at least three high-risk months (see Supplementary File 2, Table S2). An additional figure shows the fold difference between the reported and predicted incidence for each month in 2021 as a heatmap (see Supplementary File 2, Figure S5).

**Fig. 4:**
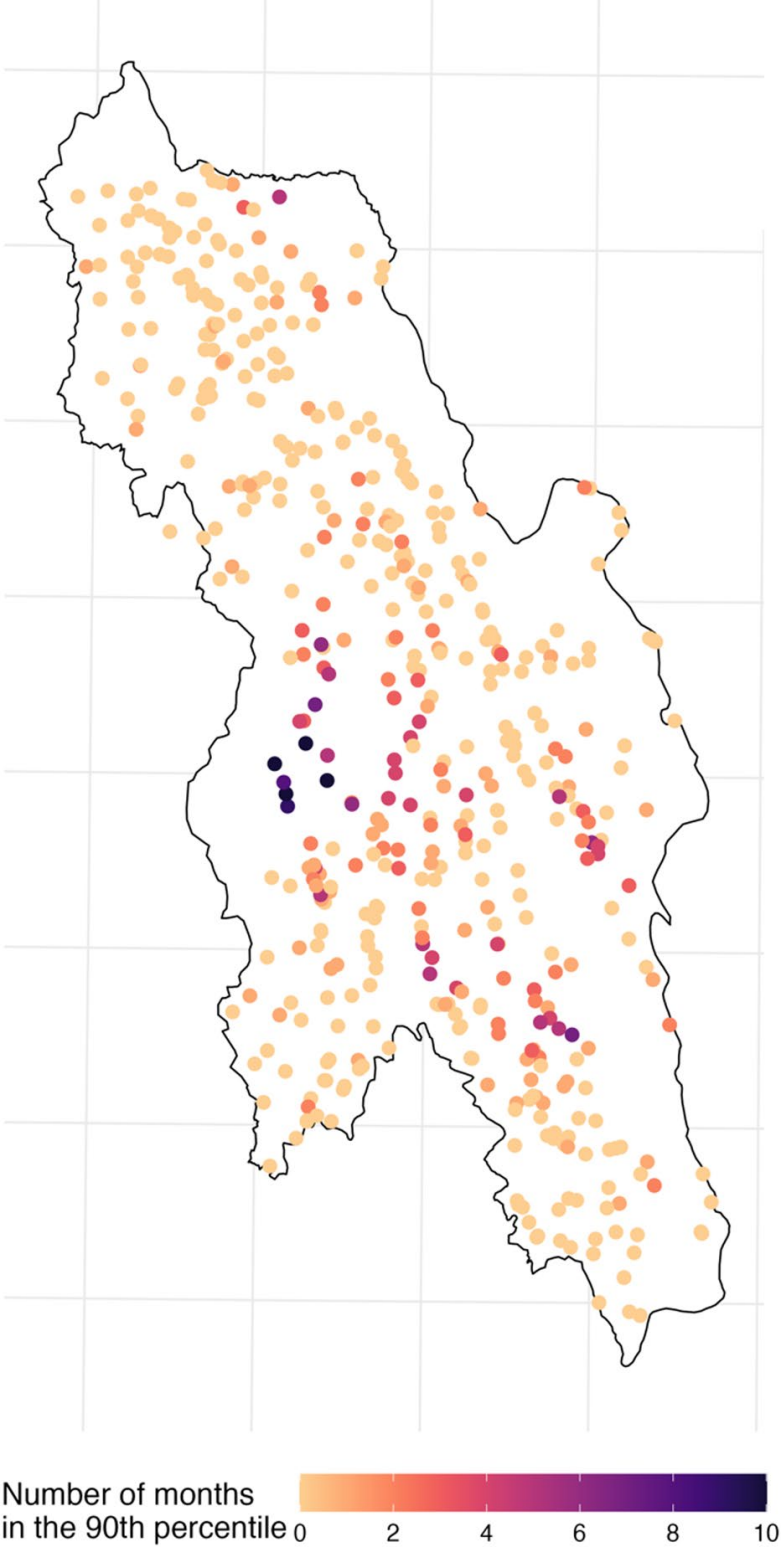
Number of high-risk months for each village. High-risk months were defined as those in which the fold difference between reported and predicted incidence corresponded to the 90th percentile or above (derived from all villages that month), indicating villages with persistently higher-than-expected *P. falciparum* incidence based on the geostatistical model.

At eight malaria posts, the difference between predicted and reported *P. falciparum* incidence was higher than for 90% of malaria posts (deemed high-risk months) for at least six months in 2021. Notably, four of these malaria posts reported 10 high-risk months in the year (Fig. 4).

**Fig. 5:**
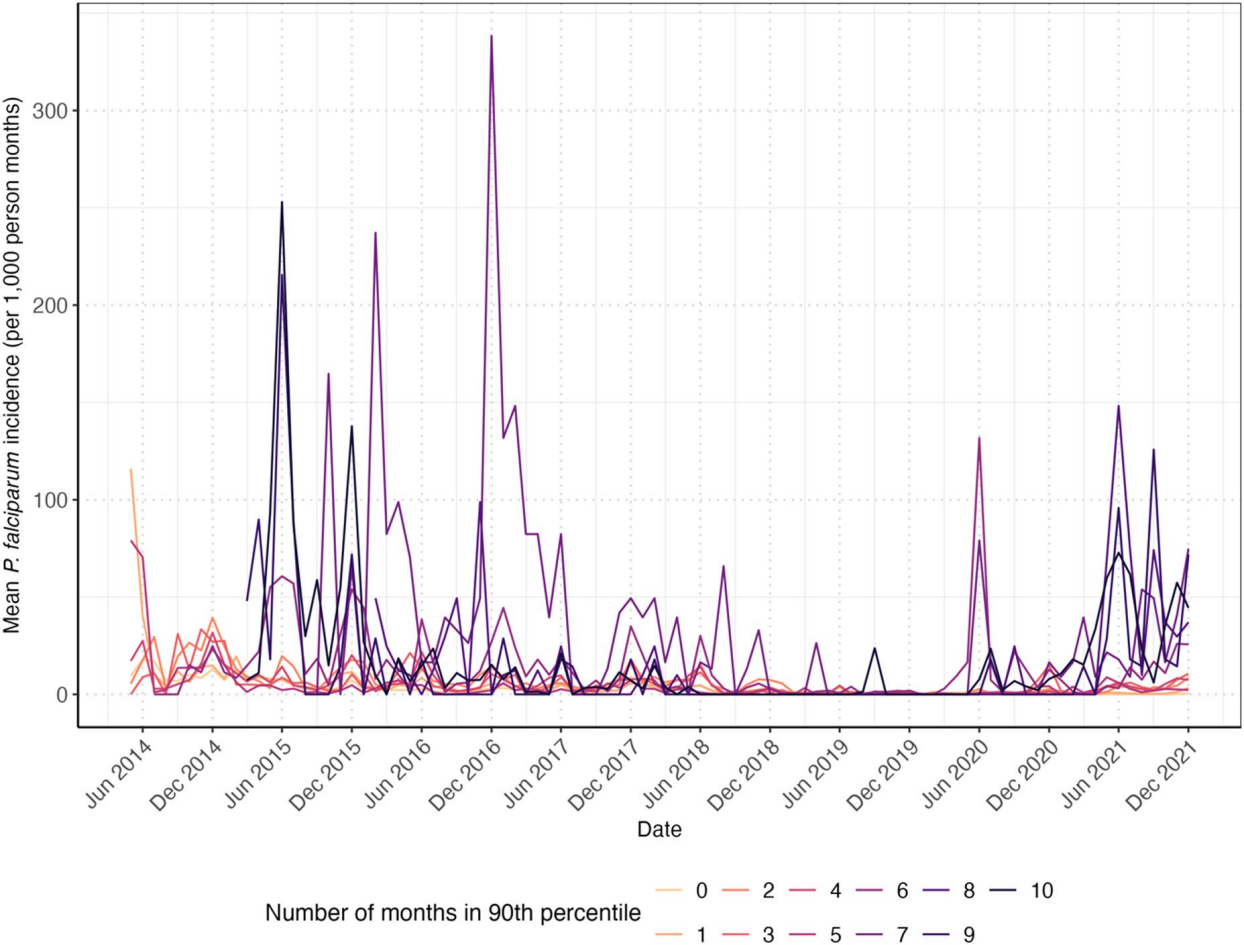
Mean monthly *P. falciparum* incidence reported by the malaria posts by the number of high-risk months. The mean monthly incidence was estimated for each malaria post over time according to the number of months in which the fold difference between reported and predicted incidence was at or above the 90th percentile in 2021. The number of high-risk months ranged from 0 (pale orange line) to 10 (dark purple line).

Malaria posts with a higher number of high-risk months in 2021 (between 7 and 10 months) (Fig. 4) also had a higher mean monthly *P. falciparum* incidence prior to the decline in *P. falciparum* incidence in 2018, when compared with malaria posts with a lower number of high-risk months in 2021 (Fig. 5).

## Discussion

Understanding malaria transmission patterns within a given area provides malaria elimination programs with the knowledge necessary to deliver targeted interventions that effectively tackle persistent transmission^24,25^. In this retrospective analysis, a geostatistical model was fitted to weekly surveillance data collected at 507 METF-operated malaria posts between 2014 and 2021. This analysis identified 12 villages where the reported incidence greatly exceeded model-based predictions in at least three months of the year. In eight of these villages, the fold-difference between reported and predicted incidence exceeded that of 90% of malaria posts (≥ 90th percentile) in at least six months of the year. These villages could have contributed disproportionately to maintaining transmission in the area^12^ and represented potential candidates for the delivery of additional targeted interventions to accelerate malaria elimination^4^.

The predicted *P. falciparum* incidence from the geostatistical model was higher than the reported incidence for the majority of malaria posts in 2021. This is because, in 2021, the overall incidence of *P. falciparum* was low, with the majority of malaria posts reporting an incidence of less than 1 case per 1,000 person-months. The tendency of geostatistical Poisson models to smooth spatial variation, resulting in the underprediction of zero counts, is a limitation of the model. The investigation of malaria posts where the reported incidence exceeds model predictions identified a cluster of villages in the central west of Hpapun Township. This cluster was a persistent high-risk area in 2021, and could be a result of the political instability in that area following the military coup in Myanmar in February 2021.

This study provides a methodological approach for identifying high-risk villages without requiring additional data collection (provided surveillance data are reliable and geolocated). The use of geostatistical models for analysing surveillance data is not a novel approach^26–28^. However, using a geostatistical model to generate point predictions of *P. falciparum* incidence over time, and comparing these predictions with reported incidence to identify fine-scale intervention targets, is a novel approach for utilising the METF surveillance data to inform timely programmatic decisions. While we used a strict 90th percentile cut-off to identify the highest-risk villages, this threshold can be adjusted based on program resources to include a larger number of villages for investigation or intervention.

To optimise the planning and delivery of targeted interventions in high-risk areas, some factors that contribute to malaria risk within and between nearby villages should be considered^29^. When nearby villages are connected, either geographically, due to human traffic routes, or due to common mosquito populations, targeted interventions deployed effectively in both villages may have a greater impact on malaria burden^30,31^.

Several factors related to malaria risk and persistence were considered in this study, including time, season, elevation, and targeted interventions. Between 2014 and 2021, there have been substantial reductions in *P. falciparum* incidence, largely because of the continued availability of early diagnosis and treatment services^16,32^. In this analysis, elevation was used as a proxy for environmental factors strongly correlated with elevation in Hpapun, including temperature, humidity, vegetation^33,34^, and vector abundance and survival^35^. Targeted interventions (MDA and MSAT) were deployed at villages in response to high *P. falciparum* prevalence or incidence, after which the incidence typically declined^6,7^. While MDA resulted in significant declines in *P. falciparum* incidence^6^, reductions in *P. falciparum* following MSAT were mostly attributed to the continued availability of early diagnosis and treatment offered by the malaria post network^7^.

The covariates included in the model account for some of the spatial correlation in *P. falciparum* incidence between malaria posts. However, due to a lack of data, not all known context-specific factors that influence *P. falciparum* incidence could be included in the model. These factors include village prevalence of asymptomatic infections, entomological data on local mosquito populations, the dates and locations of village closures in 2020 during the COVID-19 pandemic, and the extent of population displacements, along with disruptions to malaria post services caused by military attacks in Hpapun Township, which have resulted in large-scale population displacement and disruptions to malaria post service delivery. In addition, environmental variables such as vegetation and rainfall were not included in the model due to convergence issues. As a result, elevation was used as a proxy for associated environmental factors in the area. We acknowledge this as a limitation: elevation is not perfectly correlated with climatic and biophysical factors, and it cannot capture temporal changes in environmental conditions. Although seasonality is modelled through a harmonic term, the absence of time-varying environmental variables limits our ability to link seasonal patterns to specific environmental drivers. In the geostatistical model, the combined effects of these unmeasured explanatory variables are accounted for by the inclusion of a random effect for malaria post, which accounts for the non-spatially structured residual variation in *P. falciparum* incidence, and a term that accounts for the spatially structured residual variation in *P. falciparum* incidence^21^.

There are several other limitations to this analysis. First, *P. falciparum* incidence estimates were calculated based on the number of households in the village collected at the time of malaria post opening and the average number of individuals per household measured at a subset of malaria posts early in the METF program. Depending on population movement, this could result in the over- or under-estimation of *P. falciparum* incidence. However, these are the most reliable estimates of population numbers in Karen State. The reliability of incidence estimates also depends on the uptake of malaria post services; when there is a reduction in testing rates, these estimates become less reliable indicators of malaria burden. However, in the METF malaria posts, a previous study has shown that testing rates have remained relatively stable following the initial year of malaria post operation^32^. Additionally, *P. falciparum* cases in the surveillance data were diagnosed using RDTs, which have limited sensitivity and are therefore unable to detect sub-patent infections, the prevalence of which may increase with declining incidence^36^. While this means our analysis may miss areas with a high burden of sub-patent infections, the use of surveillance data still provides an accessible and practical approach for identifying high-risk areas by detecting increases in malaria incidence over time. Second, the spatial correlation between *P. falciparum* incidence across the malaria post network was modelled assuming a Euclidean (straight line) distance. While the inclusion of elevation in the model likely captures some of the variability in incidence at varying elevations, the estimation of the spatial covariance parameters could be improved by using a different measure of distance which accounts for the complex geography across Hpapun Township.

## Conclusions

Since the commencement of the METF program in 2014, *P. falciparum* incidence has declined across the program area, driven by the sustained availability and uptake of early diagnosis and treatment services at malaria posts. However, as the overall incidence has decreased, its spatial distribution has become increasingly heterogeneous. In this analysis, we identify a subset of malaria posts that in 2021 reported a disproportionate number of cases and represent persistent high-risk villages. The geostatistical model used in this study offers a practical approach for programs to detect such areas using existing surveillance data, without requiring additional resources.

## Supplementary Information

Below is the link to the electronic supplementary material.

Supplementary Material 1

Supplementary Material 2

## Data Availability

The data analysed for this study are available upon request to the Mahidol-Oxford Tropical Medicine Research Unit data access committee: (datasharing@tropmedres.ac).
