# Supplementary material for: Spatiotemporal trends in P. falciparum malaria and identification of high-risk villages in Eastern Myanmar: an 8-year observational study
Source: Sci Rep. 2025 Dec 11;16:2334. doi: 10.1038/s41598-025-32065-z (PMC12816683; doi:10.1038/s41598-025-32065-z)
Supplement: Supplementary file 1 — Supplementary Material 1 [file 41598_2025_32065_MOESM1_ESM.pdf]

Supplementary File 1: Data Visualisation

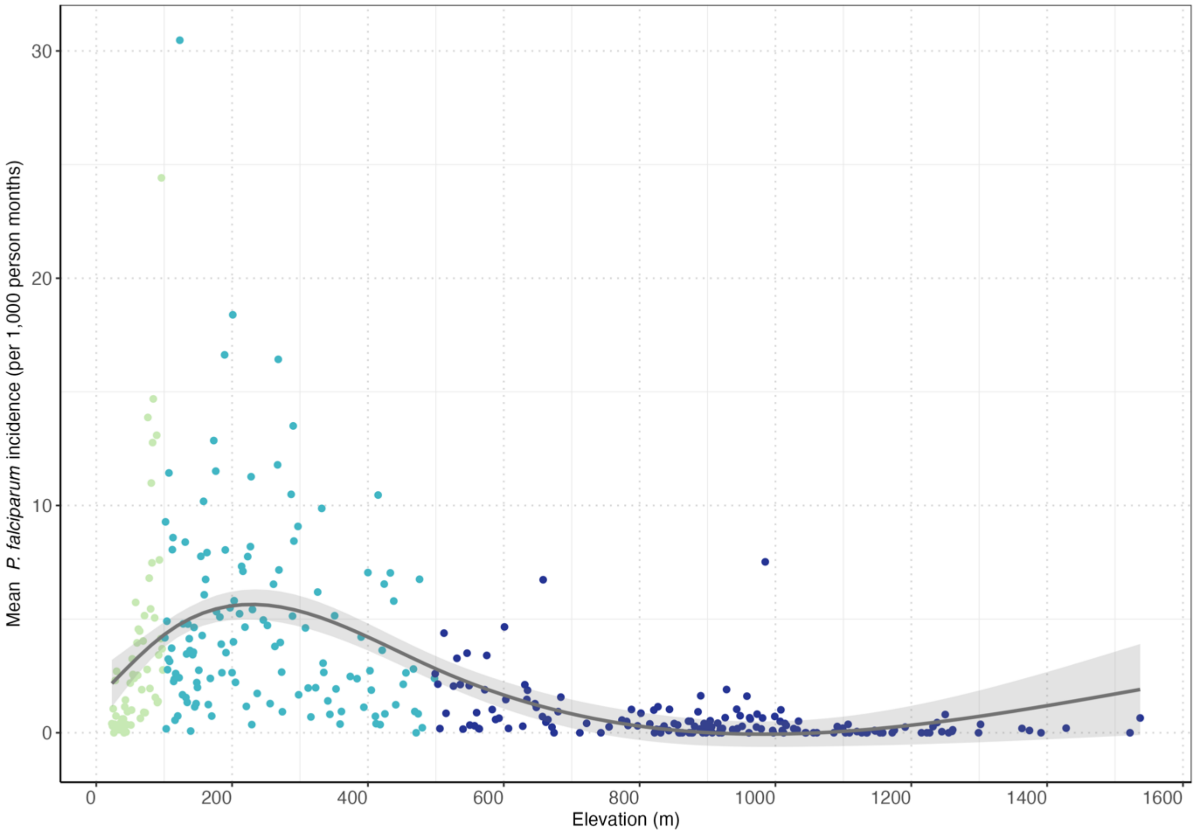

Figure S1. Relationship between elevation and monthly *P. falciparum* incidence. Each point represents the elevation of a single METF malaria post in Hpapun Township, colour-coded by spline segment: green for ≤100 m, pale blue for 100–500 m, and dark blue for ≥500 m. The grey line represents the estimated mean *P. falciparum* incidence from a Poisson mixed-effects model, which includes a random effect for malaria post and accounts for spatially structured residual variation. Shaded areas around the line indicate 95% confidence intervals.

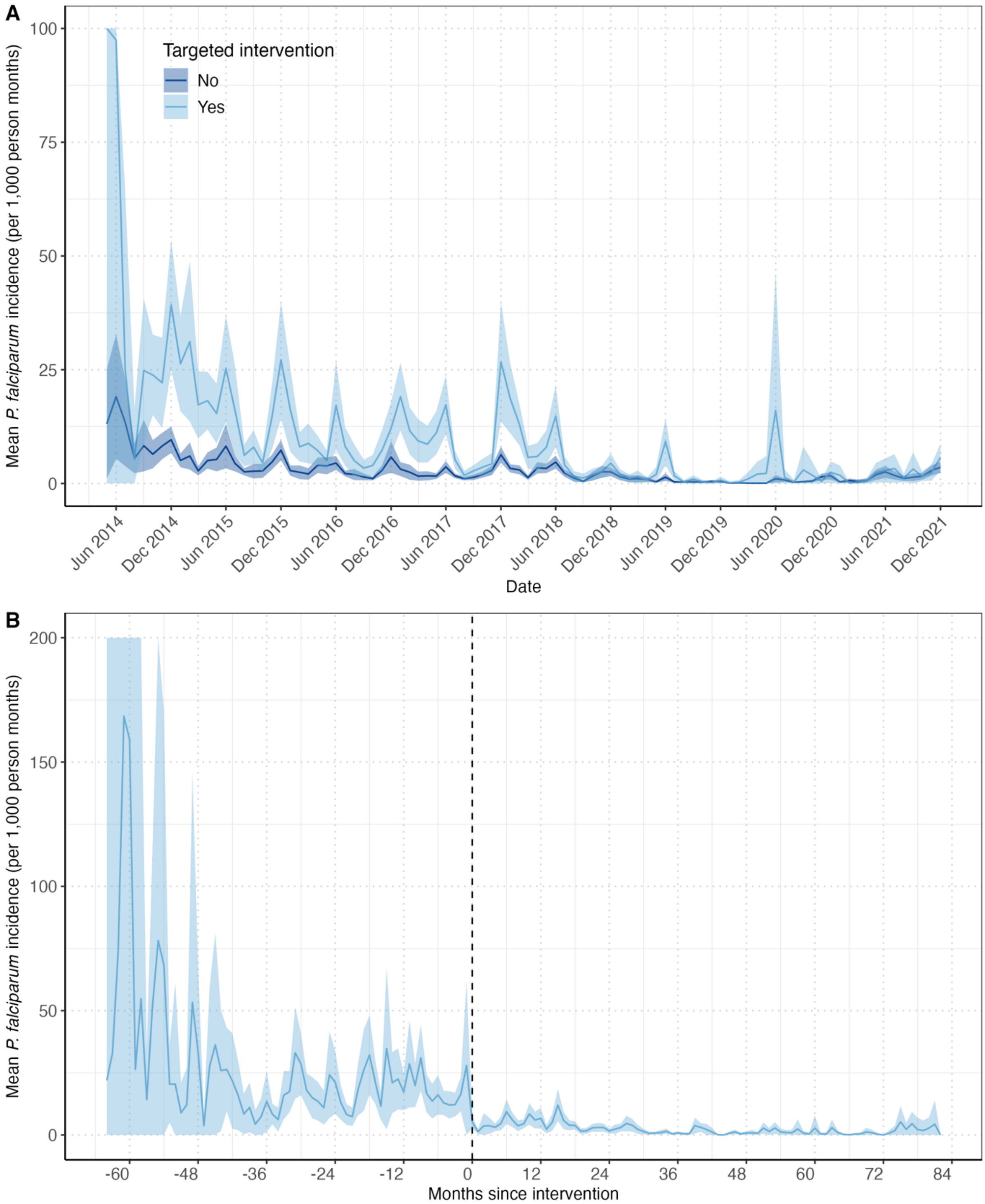

Figure S2. Mean monthly *P. falciparum* incidence by A) intervention delivery, and B) months since the intervention. A) Estimated mean *P. falciparum* incidence for malaria posts with (pale blue) and without (dark blue) a prior intervention, with corresponding 95% confidence intervals. B) Estimated mean *P. falciparum* incidence according to time in months since intervention delivery, shown as a pale blue line with 95% confidence intervals (shaded pale blue area). The upper bounds of the confidence intervals were capped at 100 (A) and 200 (B).
