# Supplementary material for: Spatiotemporal trends in P. falciparum malaria and identification of high-risk villages in Eastern Myanmar: an 8-year observational study
Source: Sci Rep. 2025 Dec 11;16:2334. doi: 10.1038/s41598-025-32065-z (PMC12816683; doi:10.1038/s41598-025-32065-z)
Supplement: Supplementary file 2 — Supplementary Material 2 [file 41598_2025_32065_MOESM2_ESM.pdf]

Supplementary File 2: Model Validation and Outputs

Testing for the presence of residual spatial correlation

To assess whether spatial correlation remains in the residuals of a Poisson mixed-effects model, we applied the *spat.corr.diagnostic* function from the PrevMap R package [22], following the method described in Diggle *et al*. [21]. This diagnostic tool evaluates the spatial dependence structure of the residuals after accounting for fixed effects and random effects in the model.

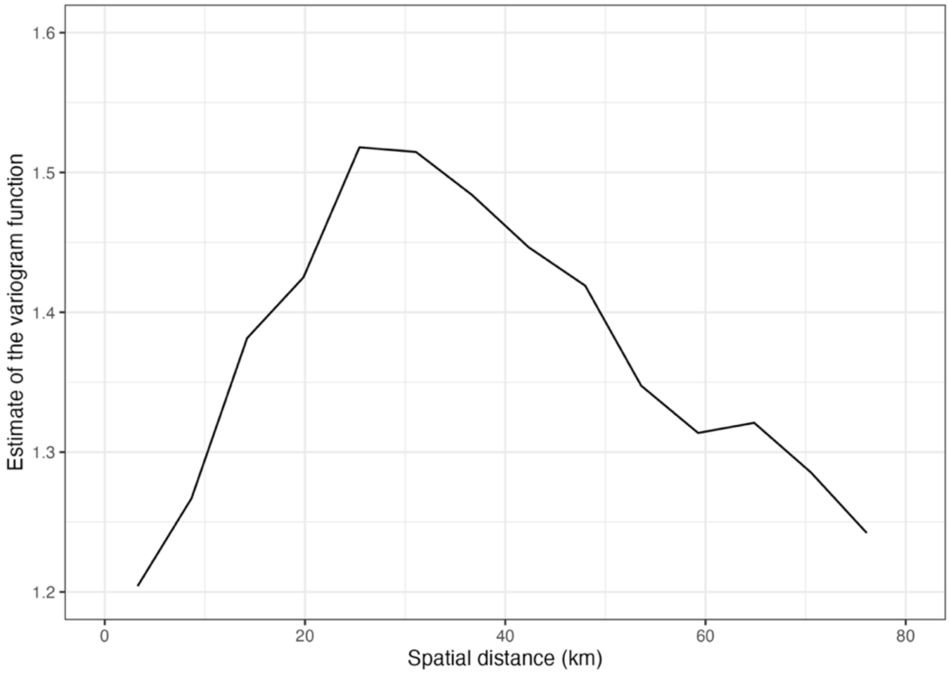

Figure S3. Empirical variogram of residuals from the non-spatial Poisson mixed-effects model. The empirical variogram was used to check for residual spatial correlation in the surveillance data collected at the METF malaria posts in Hpapun between 2014 and 2021.

Geostatistical model

Equation 1: the geostatistical model

$\log(\mu_i) = \log(\text{population}_i) + f_1(\text{time}_i) + f_2(\text{month}_i) + f3(\text{elevation}_i) + \beta_1\text{intervention}_i + \beta_2\text{intervention period}_i + S(x_i) + Z_i$

Where:

- μ_i: the expected number of symptomatic *P. falciparum* cases at malaria post i
- $\log(\text{population}_i)$: the offset term to account for the estimated village population size
- $f_1(\text{time}_i)$, $f_2(\text{month}_i)$ and $f3(\text{elevation}_i)$: smooth functions (splines/harmonics) used to model the non-linear effects of time, seasonality, and elevation, respectively
- $\beta_1\text{intervention}_i$: binary covariate indicating whether a targeted intervention was conducted at malaria post i

- $\beta_2$intervention period$_i$: binary covariate indicating whether a month was before (or, for non-intervention villages, in the absence of) or after the delivery of a targeted intervention
- $S(x_i)$: spatially structured random effect, modelled as a Gaussian process specified by an exponential correlation function, capturing the spatial correlation between malaria posts
- Z_i: the non-spatial random effect, which accounts for non-spatially referenced unmeasured heterogeneity

Table S1. Relative change in *P. falciparum* incidence in response to targeted interventions.

		IRR	95% CI
Intervention (MDA or MSAT)	No	Reference	Reference
	Yes	4.72	4.56, 4.90
Intervention period	None/Before	Reference	Reference
	After	0.26	0.24, 0.27
**Spatial covariance parameters**			
Variance of Gaussian process		0.42	0.40, 0.44
Scale parameter (φ)		14.33	12.28, 16.71
Nugget variance (τ^2)		0.17	0.16, 0.19

CI: confidence interval; IRR: incidence rate ratio.
IRR was estimated using geostatistical Poisson mixed-effects modelling. Transmission seasonality was captured using two Fourier terms per year. Natural cubic splines were used to capture the non-linear effects of calendar month and elevation on *P. falciparum* incidence.

Geostatistical model validation

To validate the geostatistical model, we assessed the spatial covariance structure by examining residual spatial correlation. This was done by computing an empirical variogram of the residuals from a version of the model where the spatially structured random effect was removed (i.e., set to zero). The variogram was computed using the *variog.diagnostic.glgm* function from the PrevMap R package [22], following the method described in Diggle *et al*. [21].

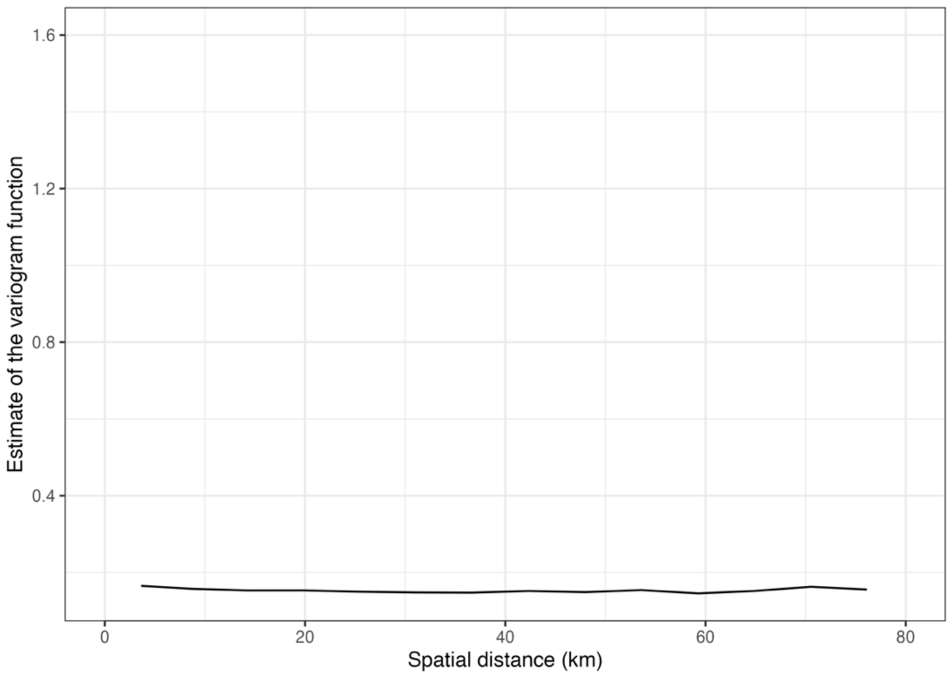

Figure S4. Empirical variogram of residuals from the geostatistical model. This variogram was used to assess residual spatial correlation in *P. falciparum* incidence measurements after fitting the model, which includes a spatially structured random effect to account for spatial dependence.

Geostatistical model predictions

Table S2. Malaria posts with at least three high-risk months.

Malaria post code	Number of high-risk months
2612	6
11013	6
11070	5
244	4
284	4
2312	4
11800	4
254	3
294	3
340	3
2662	3
11083	3

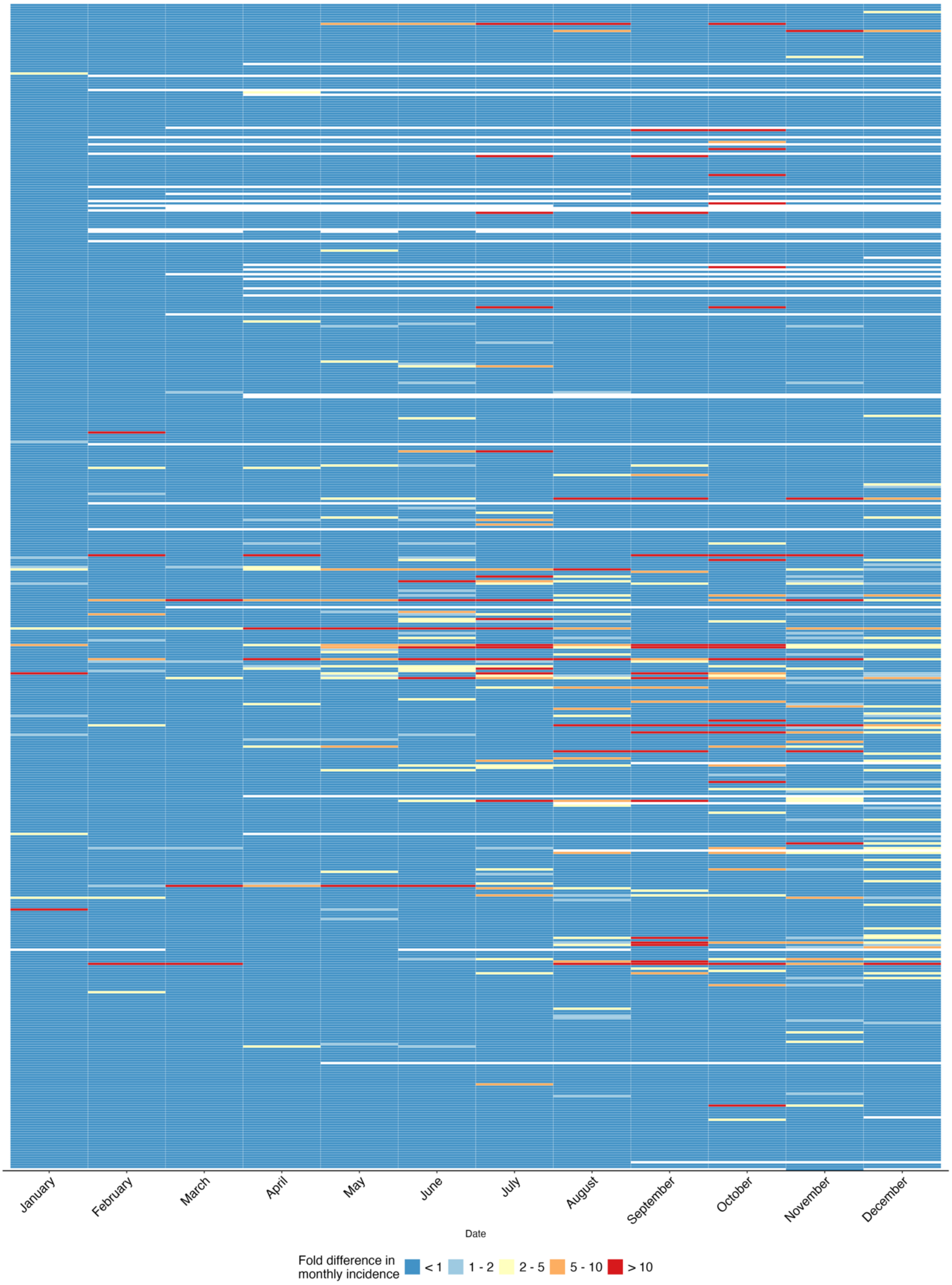

Figure S5. Fold difference between the reported and predicted *P. falciparum* monthly incidence at each village in Hpapun Township. Fold differences are shown for each village and month in 2021, ranging from dark blue (<1), indicating a lower reported incidence than predicted, to red (>10), where the reported incidence greatly exceeded the predicted values. Villages are sorted by latitude, with the northernmost malaria posts appearing on the top.
